# Understanding the colon cancer stem cells and perspectives on treatment

**DOI:** 10.1186/s12935-015-0163-7

**Published:** 2015-01-28

**Authors:** Elsa N Garza-Treviño, Salvador L Said-Fernández, Herminia G Martínez-Rodríguez

**Affiliations:** Laboratorio de Terapia Celular, Departamento de Bioquímica y Medicina Molecular, Facultad de Medicina, Universidad Autónoma de Nuevo León, León, Mexico

**Keywords:** Cancer stem cells, Chemoresistance, Colon cancer

## Abstract

An area of research that has been recently gaining attention is the relationship between cancer stem cell (CSC) biology and chemo-resistance in colon cancer patients. It is well recognized that tumor initiation, growth, invasion and metastasis are promoted by CSCs. An important reason for the widespread interest in the CSC model is that it can comprehensibly explain essential and poorly understood clinical events, such as therapy resistance, minimal residual disease, and tumor recurrence. This review discusses the recent advances in colon cancer stem cell research, the genes responsible for CSC chemoresistance, and new therapies against CSCs.

## Introduction

Colorectal cancer (CRC) is the third leading cause of cancer death worldwide, and the 5-year relative survival rate is only 8% [1] despite diagnostic and therapeutic advances. Tumor recurrence and metastasis are two critical survival-influencing factors of CRC. Many researchers have observed that some cancer cells (such as breast cancer, colon cancer, etc.) acquire the characteristics of cancer stem cells through the epithelial-mesenchymal transition (EMT) [2-4], which is responsible for promoting the invasion of CRC cells into the basement membrane and the surrounding microenvironment, including the lymph and blood vascular systems, a phenomenon that contributes to the intra- and/or extravasation of the tumor [5,6].

As a result of the EMT, the epithelial cells in a tumor, which normally interact with the basement membrane via their basal surface, are polarized, causing multiple biochemical changes, including enhanced migratory capacity, invasiveness, elevated resistance to apoptosis, and greatly increased production of extracellular matrix (ECM) components [7]. This complex process (dedifferentiation) was observed in an *in vivo* experimental model of differentiating spermatogonia, which generate germinal stem cells [8], as well as in mammary luminal cells, which convert to mammary stem cells upon the overexpression of Sox9 and Slug [9]. Chronic inflammation promotes the transition of epithelial cells to mesenchymal cells via the expression of transforming growth factor-beta (TGF-β) and tumor necrosis factor-alpha (TNF-α) [10,11].

A subpopulation of basal-like human mammary epithelial cells that show spontaneous conversion into cancer stem cell-like cells *in vitro* was recently reported [12]. It was also demonstrated in a genetic model of intestinal tumor initiation that epithelial non-stem cells can re-express stem cell markers and be converted into tumor-initiating cells. This phenomenon is strictly dependent on the degree of Wnt activation and is only observed when Wnt signaling is markedly elevated [13].

### Cancer stem cells

Evidence suggests that a small sub-population of tumor cells, termed cancer stem cells (CSCs), are responsible for propagating cancer in a highly efficient manner [14]. This malignant clonal population constitutes 0.1-10% of all tumor cells [15] of which only some have the ability to form tumors [16].

Compared to normal stem cells, CSC are thought to show no restraint with respect to cell number (i.e., proliferation); however, their slow rate of cycling plays a role in resistance to treatment (chemotherapy and radiotherapy) and tumor recurrence [17,18]. Also, the ability of CSCs to initiate new tumors may be of critical importance for metastatic colonization. In fact, the ability of a cancer cell to seed an entire tumor following experimental implantation and the ability of these cells to seed a macroscopic growth following metastatic dissemination appear to be very similar processes, leading to the notion that metastasis-forming ability is limited to CSCs [3,19].

Recently, studies have shown that growth factors such as epidermal growth factor (EGF), insulin-like growth factor-1 receptor (IGF-IR), fibroblast growth factor-2 (FGF-2), vascular endothelial growth factor (VEGF) or cytokines (TGF-β, TNF-α, IL-6) among others produced by a microenvironment can revert differentiated cells to a more stem cell – like state. Many studies have suggested that the EGF signaling pathway regulates intestinal epithelial cell and stem/progenitor cell growth and differentiation [20]. However, there is little knowledge concerning the role of growth factors in mediating proliferation and self-renewal of colon CSC.

### Properties of cancer stem cells

The properties of CSCs include infinite self-renewal potential and the capacity to differentiate into the diverse populations of cells that comprise a tumor.

- **Self-renewal** refers to the ability to form new stem cells with an identical and intact potential for proliferation, expansion, and differentiation, thus maintaining the stem cell pool. Self-renewal mechanisms that allow stem cells to persist consistently involve proto-oncogenic pathways, such as the Wnt/β-catenin and Notch pathways. Another regulator of self-renewal in the context of embryogenesis is the sonic hedgehog (Hh) signaling pathway (reported in multiple myeloma); however, little is known about the role of this pathway in adult stem cells and CSCs [21]. The preferential expression of Hh in CSCs was first published in a pancreatic cancer xenograft model [22], and evidence that the Hh pathway is aberrantly activated in a number of solid tumors, including colon cancer, has also been published [23].
  A variety of signals have been shown to promote the self-renewal capacities of colon CSCs, including the Wnt pathway and the prevention of β-catenin-dependent transcription. In addition, DLL4 stimulates Notch receptors on neighboring cells and, together with β-catenin, directs an immature transcription profile that promotes self-renewal. BMP4 is also known to counteract this self-renewal activity of CSCs by binding to BMP receptors, thereby interfering with Wnt signaling and subsequently promoting differentiation. Last, hepatocyte growth factor (HGF) has been shown to maintain colon CSCs in a stem-cell state and prevent differentiation [24].
  Homeostasis (i.e., CSC maintenance and proliferation) of the intestinal epithelium is tightly controlled and depends on the spatial organization of signals that emanate from supportive mesenchymal cells, the stromal environment, and differentiated epithelial progeny, although it remains unclear how these latter cells are integrated into the organization of intestinal cancers [25].
  Increased numbers of CSCs may occur in poorly differentiated tumors (through asymmetric cell division and damaged stem cells) as well as advanced tumors where the tumor microenvironment promotes EMT, resulting in CSC expansion. Furthermore, activation of these pathways in stem cells over the life span of an organism may predispose these cells to neoplastic transformation and homeostatic proliferation.
- **Differentiation** is defined as the ability to develop into a heterogeneous progeny of cells, which progressively diversifies and specializes according to a hierarchical process, constantly replenishing the tissue of short-lived, mature elements [26]. Recent reports about colon cancer have suggested that individual tumors, at the histopathological level, are relatively undifferentiated and may contain higher proportions of CSCs than their more differentiated counterparts, which have a significantly worse clinical prognosis [27,28].
- **Homeostatic control** is the ability to modulate and balance differentiation and self-renewal [26]. Recently, it was shown that differentiated cells in the intestinal epithelium reside in the intestinal crypts as at least two types of stem cells leucine-rich repeat containing G protein-coupled receptor 5 (Lgr5) and B lymphoma Mo-MLV insertion region 1 homolog (Bmi-1), which serve to maintain the regenerative capacities of this tissue under homeostatic conditions [29]. Lgr5 expressing cells are the more active stem cell type and serve to maintain the regenerative capacities of these tissues under homeostatic conditions. In addition, Lgr5 expressing cells are actively proliferating and extremely sensitive to Rspo1-mediated Wnt stimulation and Dkk1-mediated Wnt inhibition. In contrast, Bmi-1-expressing cells are less affected by environmental stress (i.e., not sensitive to Wnt modulation), normally quiescent and are held in reserve for “special occasions”, in which they give rise to progeny that clonally repopulate multiple contiguous crypt-villus axes during subsequent intestinal regeneration [30]. However, the homeostasis of tumoral epithelial tissues is governed by a complex program, which is controlled by niche-dependent signals that involve the subepithelial stroma (VEGF, platelet-derived growth factor, TGF-β, Nuclear factor kB), adjacent epithelial cells (Notch, Hedgedog), natural enteric flora, as well as intracellular transcription factors and the activation of signaling networks associated with the epithelium (i.e., Wnt-β-catenin). As previously reported, Wnt proteins and the Notch pathway are crucial for maintaining stem cell homeostasis, as these signals have the potential to maintain the phenotype of CSCs in the tumor mass [31]. However, the equilibrium that regulates the growth and maintenance of tumors is poorly understood.

### CSC markers

The discovery of CSC antigens is not based on the overexpression of typical tumor antigens but on the presence of antigens on populations of cells that have stem cell-like properties. However, it is important to note that variable expression levels of antigens on CSCs and their frequent co-expression on normal stem cells have made CSC antigen distinction difficult [15]. CD133, CD44, CD24, CDCP1, CXCR4, and CD26 have been identified as colon CSC surface antigens, but it is not well defined which are the best markers to identify a tumor stem cell [32,33] due to the variability found among individuals with the same tumor type [31]. A better understanding of the origin of CSCs during carcinogenesis would aid in the search for better markers [34].

### Malignant transformation of colon cancer

Tumorigenesis occurs when cells acquire six hallmarks: self-sufficiency in growth signaling, insensitivity to anti-growth signaling, evasion of apoptosis, unlimited replicative potential, sustained angiogenesis and tissue invasion. An initial event of cancer involves genetic defects that cause DNA instability activation followed by less tumor suppressor gene or gatekeeper pathway. The idea that the key tumorigenic mutations occur in a few cells that can self-renew and reside in tissues in the long-term is a major shift in thinking and has implications in the ability of adenomas to progress to carcinoma, and finally, in treatment failure [35,36].

### Model of colon carcinogenesis

**Figure 1:**
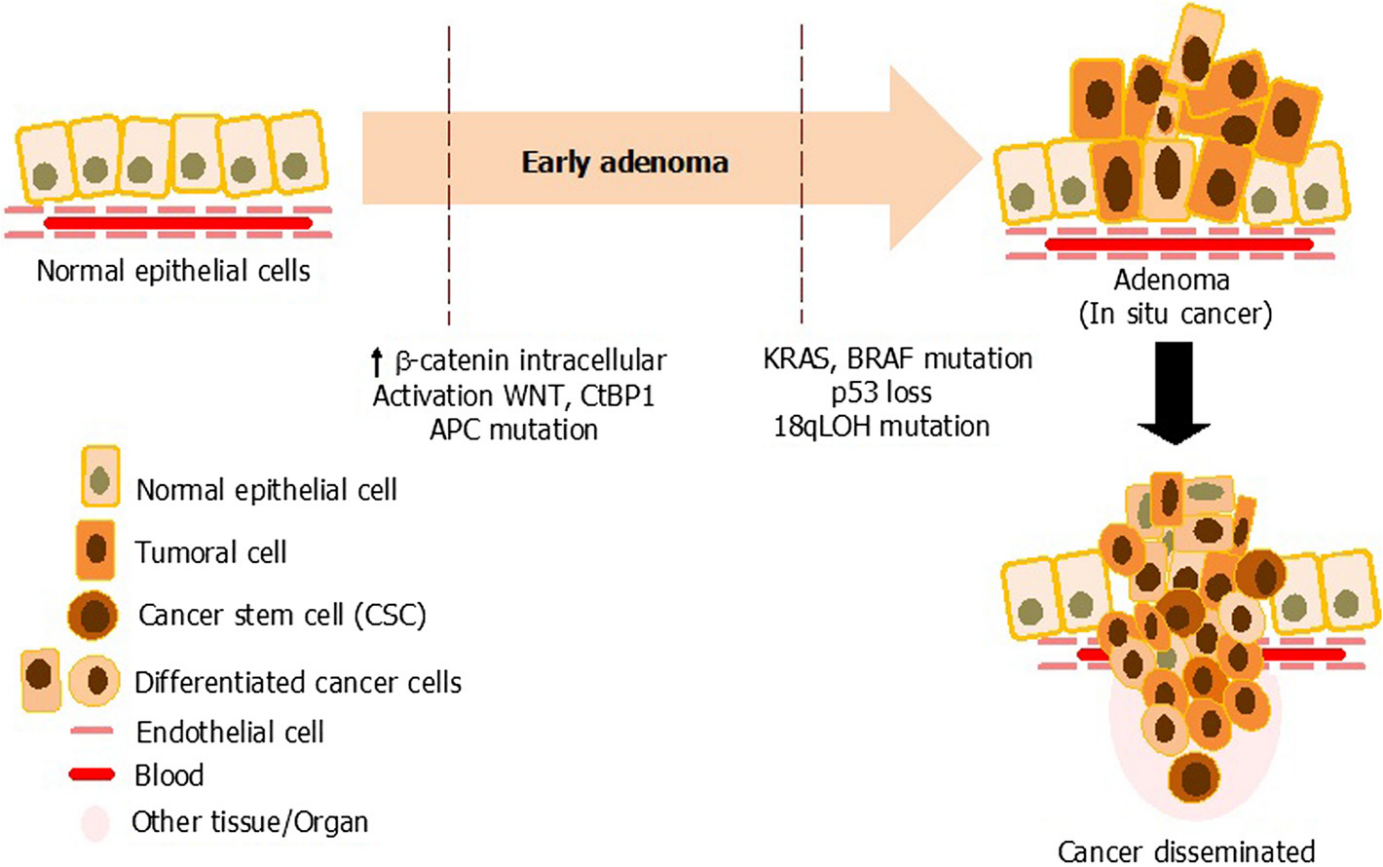
**Carcinogenesis of colon cancer.** Progression of colon normal epithelium to invasive carcinoma goes through several stages. The invasive carcinoma stage involves epithelial cells losing their polarity and detaching from the basement membrane altering cell-ECM interactions and signaling networks producing changes in stem cells that generate cancer stem cells. Malignant phase of tumor growth can progress from this stage to metastatic cancer, also involving invasion of tissue by feed blood vascular systems.

The model of carcinogenesis begins with an expression increase in intracellular β-catenin in normal colon epithelial tissue, which results in the prolonged activation of the Wnt pathway, β-catenin stabilization, and C-terminal binding protein 1 (CtBP1). In addition, APC inactivation contributes to adenoma initiation as the first step. KRAS activation and β-catenin nuclear localization act synergistically to promote the progression of adenoma to carcinoma [37]. Also, the loss of p53 and the heterozygosity of chromosome 18q [38] are frequently observed in advanced colorectal cancer (only the *TP53* mutation is generally believed to occur at the time of transition of an adenoma to cancer). Mutations in the transforming growth factor-beta receptor (TGFBR) and phosphatidylinositol 3-kinase (PIK3CA) genes are reported to be factors involved in tumor progression [39]. In addition, recent studies have indicated that the cellular origin of CRC initiation might involve the normal stem cells of the intestine, rather than progenitors or differentiated cells. It has been hypothesized that transformed stem cells progress to intestinal adenomas (Figure 1).

### Cancer stem cells and carcinogenesis

Cancer stem/progenitor cells and their progeny attain more malignant phenotypes during primary cancer progression via three distinct pathways of genomic instability: the chromosomal instability, microsatellite instability, and CpG island methylator phenotype pathways [40]. However, carcinoma develops a decade after the appearance of polyps. Without prophylactic colostomy, colon cancer appears in 100% of these patients [39].

The tumorigenic pathways cooperate to activate different downstream signaling effectors leading to carcinogenesis, including PI3K/Akt/molecular target of rapamycin (mTOR), nuclear factor-kappaB (NF-kB), mitogen-activated protein kinases (MAPKs), Myc and polycomb group (PcG) proteins, such as B lymphoma Mo-MLV insertion region 1 homolog (BMI-1). Cooperation between these signal transduction elements, in turn, plays a critical role in the high self-renewal ability, sustained proliferation, survival, invasion and metastasis of cancer stem/progenitor cells and their progeny [39,41-47].

During the transformation process, defects in the DNA mismatch repair (MMR) system result in microsatellites in the genome that are either longer or shorter than those in the parent cell. This phenomenon is termed microsatellite instability (MSI). These are present in two copies in most individuals and are responsible for 15%–20% of colon cancer cases [48]. Inactivation of MMR enzymes can occur either through the aberrant methylation of promoter CpG islands in the MLH1 gene or through point mutations in MMR family genes. The majority of these inactivation events are due to the epigenetic silencing of MLH1 gene expression by promoter hypermethylation [49-52]. Aberrant hypermethylation involves the covalent attachment of a methyl group to the 5´ position of cytosine and takes place in repetitive CG dinucleotides or CpG-rich stretches of DNA within the promoter region, resulting in transcriptional silencing. In addition, other secondary pathways that regulate cellular proliferation, differentiation, senescence, and apoptosis (RAS/RAF/MAPK) have been reported [27,39].

Crypt progenitors divide every 12–16 h, generating approximately 300 cells per crypt each day [53]. The committed transit-amplifying (TA) cells are responsible for building tissue mass. TA cells typically undergo a limited number of cell divisions, after which they terminally differentiate into either secretory (goblet, paneth and enteroendocrine) cells or enterocytes. Their proliferation is balanced by apoptosis and cell shedding at the other end of the epithelial conveyor belt, the tip of the villus. However, in the tumor process, it is assumed that CSCs originate from normal stem cells after the accumulation of mutations, and growth-promoting signals change the microenvironment or niche for CSCs undergoing uncontrolled proliferation [54,55]. In recent years, CSCs have received intense interest as key tumor-initiating cells that may also play an integral role in recurrence following chemotherapy, particularly because of their ability to proliferate [56] and self-renew [57] after chemotherapy, irradiation or both [58]. As a result, a number of mechanisms for the chemoresistance of CSCs have been identified [59].

### Chemoresistance

Treatment with CRC-based chemotherapeutic regimens principally includes 5 fluorouracil (5FU), oxaliplatin and/or leucovorin or 5-FU, leucovorin and irinotecan (FOLFIRI). However, drug failure occurs in 90% of metastatic cancers and is attributed to therapeutic resistance, which is associated with increased aerobic glycolysis, fatty acid synthesis, and glutamine metabolism, resulting in decreases in drug-induced apoptosis [60]. In addition, drug efflux transporter proteins (or ABC transporters) are generally found to be overexpressed in drug-resistant cancer cells [61].

### Cancer stem cells and chemoresistance

Chemotherapeutic drugs display antitumor effects in part by inducing oxidative damage, which increases glycolysis and results in high levels of NADPH (an antioxidant), an event that can be associated with cancer chemoresistance; however, increased ATP can activate ABC transporters to increase drug efflux [61] and upregulate HIF-1α signaling, inducing hypoxia-associated drug resistance. Specifically, HIF-1α induces the expression of genes that promote survival through anti-apoptotic signaling (survivin, Bcl-XL, Mcl-1) or other survival mechanisms, such as autophagy by BCL2/adenovirus E1B 19 kDa interacting protein 3 (BNIP3) or BCL2/adenovirus E1B 19 kDa protein-interacting protein 3-like (BNIP3L) [62-65]. Also, HIF-1α expression decreases pro-apoptotic signaling by inducing the expression of decoy receptors, such as DcR2, that compete for pro-apoptotic signaling factors, such as tumor necrosis factor-related apoptosis-inducing ligand, thereby decreasing productive signaling through apoptosis-inducing receptors, including DR4 and DR5 [66-69]. This attenuation of pro-apoptotic signaling allows cells to tolerate a higher level of chemotherapeutic insult before inducing cellular death pathways.

### Signals of chemoresistance

Another mechanism of CSC drug resistance is the preferential activation of pro-survival signaling. For example, CD44, a receptor for hyaluronan (HA), is a major marker for CSCs in a variety of cancers. The binding of CD44 by HA can lead to the association of CD44 with epidermal growth factor receptor (EGFR) [63,64]. This association activates MAP kinase and other cellular signaling pathways, promoting cell survival in response to antineoplasic treatments, such as cisplatin, methotrexate, and adriamycin [70]. Furthermore, the HA-CD44 interaction activates EGFR-elicited cellular signaling pathways without engagement of the ligand EGF [70-72], which leads to resistance to targeted anti-EGFR therapy [71].

Another signaling mechanism involves CD47. CD47 is a widely expressed transmembrane protein, a receptor for thrombospondin family members, and the ligand for signal regulatory protein alpha (SIRPα). The CD47/SIRPα interaction has been attributed as a mechanism that provides the cell with an anti-phagocytic signal. Tumor cells express high levels of CD47 to avoid phagocytosis by tumor-associated macrophages, and CD47 expression has been shown to be required for the survival, growth and metastasis of hematopoietic and solid tumors [72].

Finally, the failure of conventional treatment regimens, particularly chemotherapy [73,74] and radiotherapy [75], can be attributed to CSCs. In fact, CSCs can be segregated from a cell population by selecting for cells that exhibit resistance to standard cancer treatments [76]. New strategies are being sought to address this problem, including a chemotherapy response assay that evaluates the chemosensitivity of a tissue sample (*in vitro*) and the design of compounds against CSCs.

### Perspectives on treatment

Recently several compounds and drugs have been discovered selectively against CSC [77]. Some of these are microbe-derived and plant-derived biomolecules [78,79], small molecule inhibitors that target key components of the intrinsic signaling pathways of CSCs, some classical drugs, such as metformin, tranilast, and thioridazine [77], monoclonal antibodies (mAbs) and antibody constructs that are directed against CSC-specific cell surface molecules, such as the CD44, CD47, EpCAM, CD123, GD2, Lgr5, IGF-IR, Dll4 and FZD receptors [15,80], or antibody derivatives. Technologies such as antibody PEGylation [81] polysialylation [82] and albumin can be used to engineer a longer blood half-life for use against target signaling pathways and/or molecules that selective operate in CSCs, some of which are also capable of killing subpopulations of cancer cells that do not display CSC properties. Therapeutic approaches with mAbs [83-90], antibody constructs and novel therapeutic strategies against colon CSCs [91,92] are summarized in Table 1, and some of these methods are reviewed in detail below.

**Table 1:** **Monoclonal antibodies and nanocarriers against human colon cancer stem cells**

**Target**	**Compound**	**Class**	**Status**	**Reference**
**anti-EpCAM/anti-CD3**	MT110 (solitomab)	BITE; human recombinant single chain bispecific bifunctional mAb construct	Preclinical, *in vitro*, xenograft mice Phase I clinical study, advanced solid tumors	[82]
**anti-EpCAM/anti-CD3/Fcγ**	Catumaxomab (Removab™, TRION Pharma, Germany)	Triomab; recombinant chimeric two half antibody, each with one light and one heavy chain from mouse IgG2a and rat IgG2b isotypes. Bispecific, trifunctional mAb construct	Phase I–III clinical studies	[85]
**anti-IGF-IR**	AVE1642	Humanized recombinant IgG1 mAb, derived from mouse anti-IGF-IR IgG1mAb EM164	Preclinical, xenograft mice	[78]
	Figitumumab (CP-751,871)	Humanized IgG2 mAb	Preclinical, *in vitro*, xenograft mice	[81]
**anti-DLL4**	OMP-21 M18 (Demcizumab)	Humanized IgG2 mAb	Preclinical, xenograft mice Phase I clinical studies, combination with drugs	[79,83]
**anti-Frizzled (1, 2, 5, 7, 8)**	OMP-18R5 (vanticumab)	Humanized recombinant IgG2 mAb	Preclinical, xenograft mice	[80,86]
**Drug efflux protein multidrug resistance 1 (MDR1)**	Lipid nanocarriers	PEI-lipid nano complex with an MDR1-targeting siRNA (siMDR1)	Human colon CSC (CD133^+^ enriched cell population)	[84]
**Cancer stem-like cells (CSLCs) that are resistant to conventional chemotherapy and the bulk cancer cells**	CSO-SA/OXA micelles	Micelle formulation of oxaliplatin (OXA) encapsulated in chitosan vesicle	*In vitro* (HT29 and SW620 line cellular [CD133^+^/CD24^+^])	[87]

### Antibodies against cancer stem cells

Colon CSCs that are resistant to 5FU or oxaliplatin can be sensitized with an interleukin-4 blocking antibody. The autocrine stimulation of interleukin-4 receptors on CSCs has been suggested to contribute to their stemness, including their drug-resistant phenotype [76,93]. Another study demonstrated that the anti-EREG antibody (epiregulin, epidermal growth factor family) is efficacious against tumor metastasis [94]. This antibody showed only moderate activity against established xenograft tumors in mice NOG (NOD/Shi-scid/IL-2Rcnull) but exhibited a stronger efficacy in a metastatic model tested in this study, suggesting that the anti-EREG antibody is efficacious in the early stage of cancer development when cancers are rich in CSCs [94].

Other authors have reported the use of antibody constructs that target CSCs, which are more effective when combined with conventional cytostatic drugs [77,88]. Combinations or cocktails of antibodies against bulk tumor targets and CSC targets can sometimes destroy the whole tumor as well as the resilient CSC population, preventing relapse [95]. Also, the design of bi-specific antibodies that recognize both CSC markers (which are co-expressed on normal stem cells) and tumor antigens could be used as a novel treatment to increase the specificity of CSC targeting [96].

### Nanotechnology and cancer stem cells

Today, nanotechnology is encountered in many aspects of our daily lives. The growing field of biotechnology requires new tools that can easily interact with proteins in even smaller sizes. Nanonization can be applied to drugs for pharmaceutical use as a drug delivery system, resulting in the effective and selective delivery of treatment against tumor cells [97].

Drug delivery systems can be optimized with respect to drug extrusion, low aqueous solubility and stability, and high nonspecific toxicity using nanocarriers, such as nanoparticles (NPs), liposomes, micelles, nanotubes and nanogels, which have high penetrability. For example, polymeric micelles with a core-shell structure can be formed by the self-aggregation of polymeric amphiphile for the delivery of cytotoxic agents after intravenous administration in solid tumors providing a significant advantage against tumor by increased the enhanced permeability and retention effect of the cytotoxic compounds [97]. A novel micelle formulation of oxaliplatin encapsulated in a chitosan vesicle (CSO-SA/OXA micelles) [92] shows an excellent internalization ability that targets the tumor cell nucleus and increases the oxaliplatin concentration in tumor cells, which was shown to eliminate CSCs *in vitro* and *in vivo*.

In another example, solid lipid nanoparticles (SLNs) were utilized for the release of 5-FU inside the colonic medium for the local treatment of colon cancer; however, these SLNs have not been evaluated in an *in vivo* model to date [98]. The uptake of nanovehicles may occur via endocytosis, in which the free drug is internalized into cancer cells by molecular diffusion. Using drug-loaded nanovehicles, the drug can be efficiently delivered via penetration of the cell membrane, especially in chemoresistant tumor cells.

Liu C. has described a method by which chemotherapy resistance in colon CSC can be overcome through the siRNA-mediated knockdown of the drug efflux protein MDR1, which often is overexpressed in CSCs. Utilizing a moderate-throughput approach, the authors generated libraries of lipid nanocarriers composed of varying ratios of cationic polyethylenimine (PEI1200), polyethylene glycol (PEG) and a biodegradable lipid crosslinker such as 1,2-dilinoleyloxy-3-dimethylaminopropane (DLin-DMA). The electrostatic complexes formed by mixing with siRNA were screened for knockdown efficiency, and the optimized nanocarrier formulations were found to achieve >90% silencing. It has also been reported that treatment of colon CSCs with lipid nanocarriers containing MDR1-directed siRNA leads to efficient MDR1 knockdown and sensitizes cells to subsequent paclitaxel treatment tested in an in vitro model using CHOK1 cells [89].

In vitro assays have shown that nickel zinc ferrite nanoparticles produce cytotoxicity in epithelial cancer cells [99], and silver nanoparticles have been demonstrated to induce apoptosis in human colon cancer cells (HCT-116) dependent on the p53 expression [100]. Silver nanoparticles can be dissolved in solution, which prevents their agglomeration, or entrapped in a matrix. These kinds of particles represent interesting candidates for research as broad-spectrum bactericidal and virucidal compounds due to their effectiveness at small doses as well as their minimal toxicity and side effects [94]. Thus, the administration of silver nanoparticles (Table 1) during chemotherapy treatment in cancer patients could protect against the recurrent infections caused by chemotherapy agents. Nonetheless, conclusive safety has not been extensively demonstrated in animal models, and therefore, additional testing of silver nanoparticles is required before they can be used in clinical applications.

## Conclusion

A better understanding of how tumor-initiating cells, such as CSCs, escape chemotherapy, the establishment of appropriate biomarkers, and the definition of novel clinical endpoints for monitoring the efficacy of combined and multimodal therapeutic strategies will be a challenge to improving future colon cancer treatment.

## Abbreviations

CRC: Colorectal cancer
CSC: Cancer stem cells
EMT: Epithelial mesenchymal transition
ECM: Extracellular matrix
Lgr5: Leucine-rich repeat containing G protein-coupled receptor 5
TNF-α: Tumor necrosis factor-alpha
TNF-β: Transforming growth factor-beta
Hh: Hedgehog
HGF: Hepatocyte growth factor
CTBP1: C-terminal binding protein 1
PI3K: Phosphatidylinositol 3-kinase
Bmi-1: B lymphoma Mo-MLV insertion region 1 homolog
VEGF: Vascular endothelial growth factor
IGF-IR: Insulin-like growth factor-1 receptor
FGF-2: Fibroblast growth factor-2
TGFBR: Transforming growth factor-beta receptor
BNIP3: BCL2/adenovirus E1B 19 kDa interacting protein 3
BNIP3L: BCL2/adenovirus E1B 19 kDa protein-interacting protein 3-like
NF-kB: Nuclear factor-kappaB
MAPKs: Mitogen-activated protein kinases
PcG: Polycomb group proteins
MMR: DNA mismatch repair system
MSI: Microsatellite instability
TA: Transit-amplifying cells
5FU: 5 fluorouracil
FOLFIRI: 5-FU leucovorin and irinotecan
HA: Hyaluronan acid
EGF: Epidermal growth factor
HIF-1α: Hypoxia-Inducible Factor-1 alpha
EGFR: Epidermal growth factor receptor
NPs: Nanoparticles
SLNs: Solid lipid nanoparticles
PEG: Polyethylene glycol
EREG: Epiregulin epidermal growth factor family
mice NOG: NOD/Shi-scid/IL-2Rcnull
CSO-SA/OXA: Formulation of oxaliplatin encapsulated in a chitosan vesicle
DLin-DMA: 1,2-dilinoleyloxy-3-dimethylaminopropane
